# Trade-offs between tRNA abundance and mRNA secondary structure support smoothing of translation elongation rate

**DOI:** 10.1093/nar/gkv199

**Published:** 2015-03-12

**Authors:** Thomas E. Gorochowski, Zoya Ignatova, Roel A.L. Bovenberg, Johannes A. Roubos

**Affiliations:** 1 DSM Biotechnology Center, P.O. Box 1, 2600 MA Delft, The Netherlands; 2 Department of Biochemistry, Institute of Biochemistry and Biology, University of Potsdam, 14476 Potsdam-Golm, Germany; 3 Biochemistry and Molecular Biology, Department of Chemistry, University of Hamburg, 20146 Hamburg, Germany

## Abstract

Translation of protein from mRNA is a complex multi-step process that occurs at a non-uniform rate. Variability in ribosome speed along an mRNA enables refinement of the proteome and plays a critical role in protein biogenesis. Detailed single protein studies have found both tRNA abundance and mRNA secondary structure as key modulators of translation elongation rate, but recent genome-wide ribosome profiling experiments have not observed significant influence of either on translation efficiency. Here we provide evidence that this results from an inherent trade-off between these factors. We find codons pairing to high-abundance tRNAs are preferentially used in regions of high secondary structure content, while codons read by significantly less abundant tRNAs are located in lowly structured regions. By considering long stretches of high and low mRNA secondary structure in *Saccharomyces cerevisiae* and *Escherichia coli* and comparing them to randomized-gene models and experimental expression data, we were able to distinguish clear selective pressures and increased protein expression for specific codon choices. The trade-off between secondary structure and tRNA-concentration based codon choice allows for compensation of their independent effects on translation, helping to smooth overall translational speed and reducing the chance of potentially detrimental points of excessively slow or fast ribosome movement.

## INTRODUCTION

Translation of mRNAs into protein is crucial for cell viability and function and proceeds at a non-uniform rate along transcripts (1). While much focus has been placed on the translation initiation step that is often rate limiting for endogenous genes (2,3), there is growing realization that the variable dynamics of translation elongation also play a crucial role in both fine-tuning expression levels and ensuring the correct folding of soluble proteins (4–6). Shifts in translational resources such as tRNA concentration and charging, dramatically affect translational efficiency (7–9) and have been suggested as a clear biomarker in diseases such as breast cancer (10). Synonymous substitutions that have long been thought to be neutral for protein folding, are increasingly recognized as deleterious for protein biogenesis (11,12). Furthermore, synonymous codon usage strongly influences translational efficiency. Optimization of synonymous codon choice when designing recombinant genes, through minimization of rare codon use in the expression host, in many cases leads to significantly increased protein yields for mostly single-domain heterologous proteins (13,14).

**Figure 1.**
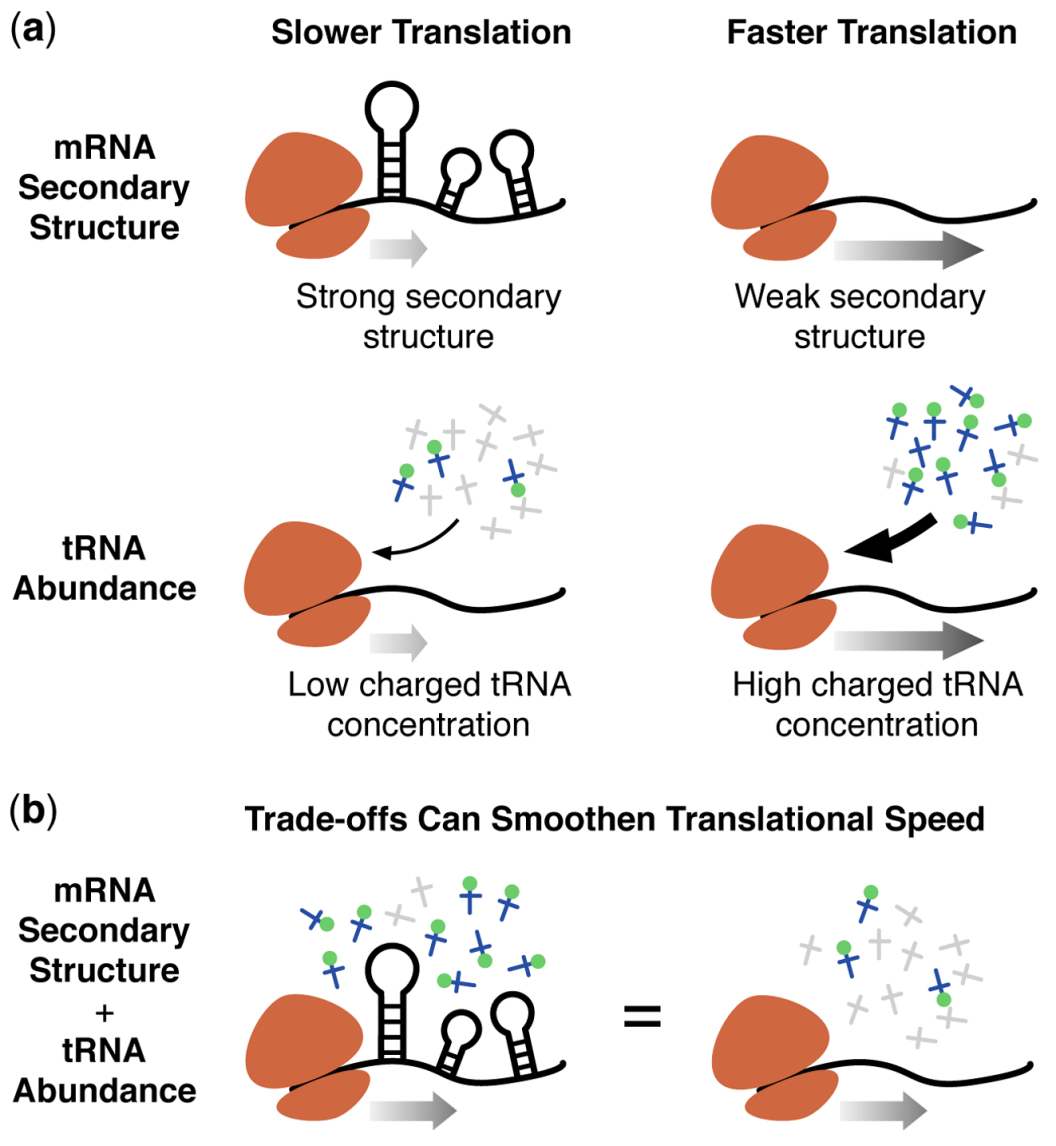
Trade-offs between mRNA secondary structure and tRNA abundance can potentially smoothen overall elongation rate. (**a**) mRNA secondary structure and cognate aa-tRNA abundance each affect elongation speed. (**b**) By trading-off the negative effect of one factor with the positive effect of the other, a more constant elongation rate can be maintained along a transcript, reducing the chance of excessively slow or fast regions.

The rate of translation of a single codon is determined by the speed of delivery of the translationally competent tRNA (i.e. aminoacylated tRNA by its cognate aminoacyl-tRNA synthetase and complexed with the elongation factor in its GTP-bound form) to the A-site of the ribosome and subsequent decoding events that take place at the ribosome. These include tRNA accommodation, peptide bond formation and translocation (15). The decoding rates of single codons vary by a factor of two (16), with proline-decoding codons being the slowest (17). However, these variations only marginally influence the elongation speed *in vivo* (16,18). In contrast, tRNAs vary in their cellular concentrations by up to 10-fold (19). Hence, a key determinant of ribosomal speed during translation is the availability and delivery of the charged cognate tRNAs to the ribosome (8,9,20). Codons pairing to high-abundance tRNAs are translated faster than codons read by low-abundance tRNAs. Models capturing this relationship successfully predict points of slow translation where ribosomes potentially pause, and have been validated using single protein studies (4,21). Clustering of slow-translating codons do not solely represent potential sites to attenuate ribosome progression along mRNA. The significant role of biophysical interactions between the ribosome, Shine-Dalgarno like sequence motifs (22), mRNA secondary structure (23–25) and the charge of the nascent amino acid chain (26) can also slow down the elongating ribosomes. A fundamental limitation of all these studies is that the potential aspects influencing translational speed are considered in isolation, neglecting the possibility of multiple factors acting in a coordinated manner (Figure 1). Surprisingly, although detailed single protein experiments have shown the significant influence of each of these factors, recent *in vivo* genome-wide ribosome profiling studies found neither in isolation as a good general predictor of ribosome speed (26–28), suggesting a competing effect between these factors. Thus, investigating each factor independently will not yield coherent results. Only integration of all contributing factors will yield a comprehensive picture of the control of the elongation process.

In this work we investigate the relationship between tRNA abundance (4,8,9) and mRNA secondary structure on the speed of translation elongation (Figure 1a). By assessing the tRNA abundance of codons in strongly and weakly structured regions of mRNAs, we find a clear relationship that suggests these factors act in an opposing manner on translational speed, potentially canceling out their individual effects. We reveal that this feature holds across both prokaryotes (*Escherichia coli*) and lower eukaryotes (*Saccharomyces cerevisiae*) with differing codon biases, also suggesting that the relationship is likely exploited by many organisms. Moreover, using randomized genome models and experimental expression data from a synonymous codon variant gene library, we illustrate selection for this feature and the beneficial effect of this trade-off on gene expression.

Together these findings highlight that while studying features influencing translation elongation in isolation is essential to gain clear mechanistic understanding, when considering such processes at a genome-wide scale and in the context of living cells, contributions of all potential factors must be integrated to gain a comprehensive understanding of their coordinated actions.

## MATERIALS AND METHODS

### Sequence data and filtering

We applied the described methods to all coding sequences of *E. coli* K-12 (GenBank accession number: NC_000913.2) and *S. cerevisiae* (GenBank accession number: GCF_000146045.1). Nucleotide sequences encoding untranslated RNAs were excluded from the analysis. To ensure that other features related to translation initiation and termination did not influence our results (29,30), the first and last 51 bp (17 codons) of all coding regions were removed before analysis. Highly expressed genes for *E. coli* (*N* = 255) were taken from the Highly Expressed Genes Database (HEG-DB) (31).

### mRNA secondary structure

Experimentally determined secondary structure for mRNAs in *S. cerevisae* were taken from a recent study (32). *E. coli* mRNA secondary structure was predicted computationally by calculating the minimum free energy of a 101 bp centered sliding window using the Vienna RNAfold software (33). Unless otherwise stated, a minimum 20 bp of consecutive high (PARS score > 0 for experimental data or predicted local free energy < −35 kcal/mol) or low (PARS score ≤ 0 for experimental data or predicted local free energy > −20 kcal/mol) secondary structure was required for that region to be included in the analysis.

### Estimating translation elongation speeds

To predict the local translational speed at each codon along a transcript, we employed the same method as Zhang *et al*. (4,21), making use of previously developed computational tools (7). This approach assumes that translation elongation is limited by diffusion of a cognate aa-tRNA to the ribosome. Thus, the instantaneous speed of a codon is proportional to the concentration of the cognate aa-tRNA pool. To account for potential local effects, for example clusters of slow codons having a greater effect on translation in that region, instantaneous speeds were smoothed using a sliding window of 27 nt, which corresponds to the approximate footprint of the ribosome (21). Predicted codon speeds were taken from two studies: Reuveni *et al*. for *S. cerevisiae* (34) and Zhang *et al*. for *E. coli* (4). Full details of the exact translation times can be found in Supplementary Table S1.

### Comparison of codon translation time distributions

**Figure 2.**
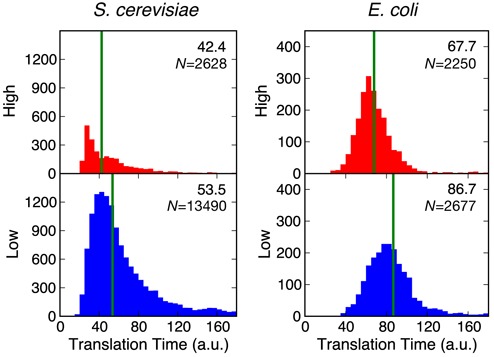
Distributions of predicted codon translation times for protein coding regions of mRNAs in the *Saccharomyces cerevisiae* and *Escherichia coli* genomes. Upper plots (red) show regions with high mRNA secondary structure and lower plots (blue) regions with low mRNA secondary structure. All analyzed sequences had a minimal region length of 20 bp. Green vertical lines and the value in top right corner denote the median value for the distribution. The shifts in codon translation times are statistically significant in both cases, see Table 1 for details.

Distributions of codon translation times for both *S. cerevisiae* and *E. coli* where found to not be Gaussian in shape (Figure 2; Table 1). Therefore, to avoid potential biasing of summary statistics, median values were used to capture general translation times and non-parametric statistical tests (e.g. Mann–Whitney) were applied for comparisons between distributions.

**Table 1.** Codon translation times for protein coding regions of high and low mRNA secondary structure across the *S. cerevisiae* and *E*.*coli* genomes

		Regions		Median time (a.u.)			
Organism	*R*_*l*_	High	Low	High	Low	Δ*t*	*P*
*S. cerevisiae*	30	111	1237	44.5 (30.5, 57.4)	57.8 (45.9, 78.3)	13.3	2.9 × 10^−11^
	20	2628	13 490	42.4 (30.0, 64.2)	53.5 (43.1, 78.7)	11.1	2.9 × 10^−102^
	10	11 253	42 011	42.1 (30.0, 67.7)	50.2 (35.9, 73.6)	8.1	1.1 × 10^−137^
*E. coli*	30	1403	1709	68.1 (59.5, 83.0)	89.8 (75.2, 159.1)	21.7	1.1 × 10^−105^
	20	2250	2677	67.8 (58.1, 83.1)	86.7 (71.6, 115.3)	18.9	1.4 × 10^−127^
	10	4613	4994	66.6 (54.4, 83.3)	82.9 (66.0, 107.2)	16.3	8.7 × 10^−166^

*R_l_* denotes the minimum region length in base pairs and Δ*t* the time difference between the median codon translation times in regions of high and low mRNA secondary structure. Median times are shown with their interquartile range in parenthesis. *P*-values are calculated using a non-parametric Mann–Whitney test.

### Functional enrichment analysis

Functional enrichment was performed using the AmiGO term enrichment tool (35). All protein coding sequences from *S. cerevisiae* were chosen as the background set for comparison and default values were used for the analysis: ‘use IAEs in calculation’ = yes, ‘maximum *P*-value’ = 0.01, ‘minimum number of gene products’ = 2.

### Gene sequence randomizations

Two randomization methods were used to destroy potential biases in synonymous codon choice and position within individual genes, (i) synonymous codon shuffling: all synonymous codons within a gene were randomly shuffled to generate genomes in which codon usage and the amino acid sequence/ordering were maintained for each gene, but the synonymous codon ordering was lost and (ii) amino acid shuffling: each codon position within a gene was shuffled uniformly at random to generate genomes with the same codon bias for each gene, but with different amino acid sequence. For each of these methods 100 randomized genomes were generated and secondary structure and tRNA abundance profiles produced as described above. These formed the null-model distributions used for comparison to assess selective pressures related to synonymous codon choice and amino acid ordering.

### Expression data for Φ29 DNA polymerase synonymous codon gene library

To assess how differences in the relationship between codon choice and mRNA secondary structure affected translation, we used absolute expression data from a library of Φ29 DNA polymerase synonymous codon variants (36). In total, this contained 39 gene variants of which 30 exhibited measurable expression. Hence, only these 30 variants were included in our analysis to avoid other factors that may have led to lack of expression. In addition, mRNA secondary structure for these variants was predicted using the same method as described above.

## RESULTS

### Fast-translated codons are enriched in regions with high secondary structure propensity

To assess whether potential trade-offs are made between tRNA abundance and mRNA secondary structure, we first focused on *S. cerevisiae* for which experimental *in vivo* mRNA secondary structure measurements are available (32). We selected regions with consecutively high and low mRNA secondary structure by considering the PARS score at each nucleotide (threshold of >0 for high and ≤0 for low secondary structure) and compared the average translation time per codon based purely on tRNA abundance taken from Reuveni *et al*. (34). We chose regions with a minimal length (initially 20 bp) as single nucleotides in isolation, even those with high propensity to be involved in secondary structure, cannot slow down ribosomes. This ensured that any localized effects that may manifest away from the codon being considered would still be captured during the analysis and enabled us to focus on regions where such effects would have most influence. In addition, to reduce other confounding effects due to initiation and termination, such as codon bias to reduce mRNA secondary structure and facilitate translation initiation (3,30), we further excluded the first and last 51 bp of all protein coding regions.

**Figure 3.**
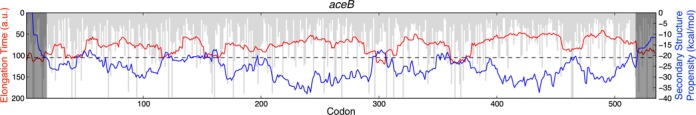
Elongation speed and secondary structure propensity are anti-correlated as exemplified by the *aceB* gene in *Escherichia coli*. Codon elongation times are calculated using the method of Zhang *et al*. (4). Light gray vertical bars represent the individual codon elongations times and the red line the smoothed translational profile. Dips correspond to regions of slow translation. The dotted horizontal line represents a threshold under which translational pausing is thought to occur (4). mRNA secondary structure predicted using Vienna RNAFold is shown by the blue line. Lower values correspond to stronger secondary structure. The first and last 17 codons are removed from our analysis (dark shaded regions).

Comparison of the estimated codon translation times (based on tRNA abundance) for regions of high and low secondary structure showed a significant difference (*P* < 2.9 × 10^−11^, Mann–Whitney test; Figure 2; Table 1). For regions with high structure we observed a bias toward codons with higher tRNA abundance and therefore shorter translation times. In contrast, regions with low secondary structure tend to be enriched in codons with low-abundance tRNAs resulting in longer translation times. This is also clearly evident in many gene traces that show an anti-correlation between these two features (Figure 3).

To ensure that this relationship was not limited to *S. cerevisiae*, we performed similar analyses with *E. coli*. This prokaryote exhibits a very different codon bias, enabling us to assess the generality of this relationship. Experimental mRNA secondary structure data is not yet available, thus local mRNA secondary structure profiles were predicted using the Vienna RNAFold software (33), which has shown a good resemblance of fit to *in vivo* data (37). Similar to *S. cerevisiae*, we observed the same clear relationship between tRNA abundance and mRNA secondary structure (Figure 3; Table 1).

An interesting difference between the two organisms was the shape of the codon translation time distributions for high and low structured regions (Figure 2). The *S. cerevisiae* data shows a highly right-skewed distribution with a large number of regions with slow translation times. This difference may be due to the greater diversity in the tRNA modifications, particularly in the anticodon loop, which would lead to a broader range of potential codon translation times that are not captured by our fixed estimates calculated from tRNA abundance. This skew is greatly reduced for *E. coli* with the distributions displaying a more normal Gaussian shape (Figure 2). The precise reason for this difference is unclear, however, it may relate to the smoothing applied to the *E. coli* data during computation of local secondary structure.

Another clear difference between the distributions for each organism was the total number of high and low structured regions (Figure 2; Table 1). *E. coli* displayed a similar number of high and low structured regions, whereas *S. cerevisiae* had much lower numbers of high structured regions in comparison to low structured regions. A potential reason for this difference is the G/C content for each genome. Analysis of all coding regions revealed G/C content of 40.1% for *S. cerevisiae* and 52.6% for *E. coli*. As G/C residues have a greater chance of forming secondary structures, the reduced number of highly structured regions in *S. cerevisiae* is likely due to this sequence bias.

In addition to these general differences, we found that the *S. cerevisiae* distribution for low structured regions displayed a weak bimodal shape. To better understand whether the low structured regions with particularly long translation times had some functional significance, we carried out GO enrichment analysis (Supplementary Table S2). This gene group was significantly enriched in GO terms related to membrane and transporter roles. This is interesting because translational elongation dynamics can play an important role in protein biogenesis including folding of local structural elements and membrane insertion (38) and translocation (39).

Next, we reasoned that in longer stretches with higher secondary structure propensity codons pairing to high-abundance tRNAs might be selected to counteract the effect of the secondary structure. To test this hypothesis, we repeated the previous analysis for both organisms over a range of minimal region length cut-offs from 10 to 30 bp. Comparing the difference in average predicted codon translation time between the high and low structured regions we again observed significant differences (*P* < 8.54 × 10^−15^, Mann–Whitney test) and a clear enrichment of fast translated codons as the minimal region length cut-off increased (Table 1). For *S. cerevisiae* this resulted in a 64% and for *E. coli*, a 33% increase in the median translation times when comparing region cut-off lengths of 10–30 bp. While the large spread of the underlying distributions (Table 1) means that these changes are unlikely to be statistically significant, in both organisms the general trends support the idea that selection will act more strongly on regions with higher secondary structure propensity, which in turn requires much greater differences in tRNA abundance to counter act it.

To test whether other gene features might influence the observed trade-offs, regions were binned into sets based on the genes in which they were found and similar comparisons of codon translation times for high and low structured regions performed. First, we considered highly expressed genes as more likely candidates for increased selection for efficient translation. Again we found a presence of a significant trade-off (*P* = 1.04 × 10^−7^, Mann–Whitney test), with an overall shift to shorter average codon translation times in regions with low and high secondary structure propensity due to a strong bias toward more abundant synonymous codons (Supplementary Figure S1–S2). This accounts for highly expressed genes showing increased usage of tRNAs with high availability, which in our model will lead to reduced translation times.

Second, we analyzed the potential influence of gene length by partitioning genes into short (<500 bp), medium (500–1500 bp) and long (>1500 bp) lengths. Notably, we observed significant trade-offs in all categories, but no clear relationship between gene length and codon translation time for high and low structured regions (Supplementary Figure S3).

**Figure 4.**
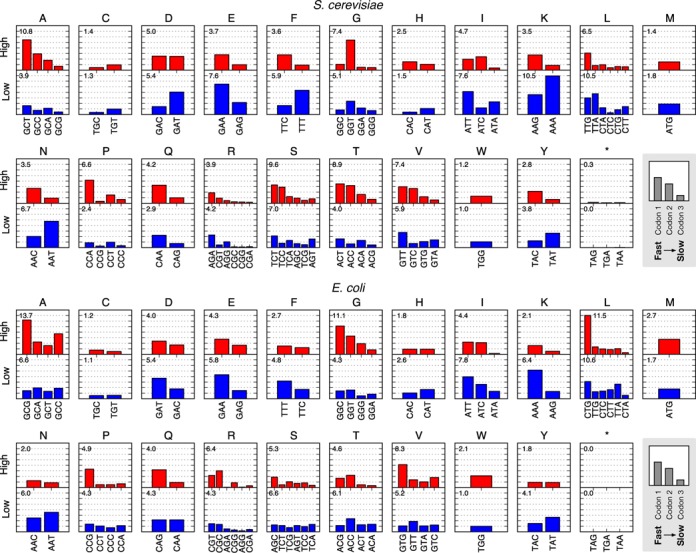
Codon usage for protein coding regions of mRNAs with high and low mRNA secondary structure across the *Saccharomyces cerevisiae* and *Escherichia coli* genomes. Separate plots are displayed for each amino acid, with upper plots (red) showing regions with high mRNA secondary structure and lower plots (blue) regions with low mRNA secondary structure. Each plot displays the normalized usage of each codon for the associated amino acid on a scale from 0 to 0.08 and codons are sorted from left to right in terms of predicted translational times, see Supplementary Table S1 (stop codons are excluded), fastest (left) to slowest (right). Notice a clear bias toward faster codons in regions with high secondary structure that is not always related to G/C content in the second and third positions (e.g. in *S. cerevisiae* see the CCA codon for proline and GAA codon for glutamic acid, both of which display a strong bias in highly structured regions even though other synonymous codons with greater G/C content exist they are, however, predicted to be the fastest translated). Horizontal grid lines are positioned at intervals of 0.01. Number in top left corner of each plot denotes the percentage of individual codons shown in the plot in relation to the total number of codons contained within the total associated low or high mRNA secondary structure regions. For example in *E. coli*, codons coding for alanine in low secondary structure regions correspond to 6.6% of all codons in low structured regions, while the same codons in high secondary structure regions correspond to 13.7% of all codons in high structured regions.

Finally we assessed the amino acid composition of high and low structured regions (Supplementary Figure S4). We found a slight enrichment of some amino acids in high or low structured regions. Alanine was commonly found in high structured regions and lysine, asparagine, phenylalanine and isoleucine in low structured regions across both organisms. This may be partially due to the sequences of the codons encoding each amino acid. For example, amino acids found in high-structured regions are coded for by codons that are G/C rich, while amino acids found in low-structured regions are coded for by A/T rich codons (Figure 4).

### Codon bias differ between highly and lowly structured mRNA regions

To better understand the specific codon choice in regions of high and low mRNA secondary structure we separately analyzed these groups and compared synonymous codon usage for each amino acid (Figure 4). Note that codons for each amino acid have been ordered from left to right in accordance to their tRNA abundance, i.e. with fast (left) to slow (right) predicted translation times.

Similar to our previous analysis, we detected a clear bias toward codons with shorter translation times (higher tRNA abundance) in the highly structured regions. This bias is less prominent or lost for the low structure regions. In many cases for the experimental structural data of *S. cerevisiae*, amino acids coded by a pair of synonymous codons, e.g. phenylalanine (F), histidine (H), lysine (K), asparagine (N) and tyrosine (Y), showed complementary ratios. More interestingly, the biases we see in many cases are proportional to the predicted translation time and therefore tRNA abundance. This is evident for the majority of amino acids with many synonymous codons, e.g. alanine (A), arginine (R), serine (S), threonine (T) and valine (V) for *S. cerevisiae*, and glycine (G) and leucine (L) for *E. coli*.

Codon choice and mRNA secondary structure are not completely separable factors with changes to one potentially influencing the other. The G/C composition of a codon influence its propensity to form a secondary structure. We observed a tendency for highly structured regions to be enriched with codons of higher G/C content. However, this relationship does not hold in all cases. For example, the most abundant codon found in highly structured regions for *S. cerevisiae* is GCT, even though codons GCC and GCG that code for the same alanine amino acid could potentially be used.

Notably, there were also several codons that displayed unusual characteristics. The GGC glycine codon in *S. cerevisiae* exhibited very low usage across both high and low structured regions even though it is read by major tRNA and is thus predicted to have very high translation rate. This may be due to the large number of modifications that *S. cerevisiae* makes to tRNAs which may result in the actual translation time for this codon being much slower than that predicted from its tRNA abundance (40). Furthermore, we observed preferences for faster translating codons in low-structured regions for other amino acids. Specifically, in *S. cerevisiae* for glutamic acid (E) and glutamine (Q) and in *E. coli* for aspartic acid (D), glutamic acid (E), phenylalanine (F) and lysine (K). While at a first glance this appears to go against our hypothesis of trade-offs helping to smooth translational speed, it is important to note that the sequence as a whole and not a single codon determine the propensity for a codon to be involved in secondary structures and that in many cases these may not be sufficiently large to interfere with translation. Of the amino acids highlighted above, all are encoded by two synonymous codons and are preferably located in low structured regions due to their A/T content, specifically at the third position.

### Genome randomizations show selection for trade-offs between mRNA structure and tRNA abundance

The biases we observed in codon usage across regions of high and low secondary structure, could merely be the result of specific codons having a greater propensity to form strong structural motifs. Thus, next we generated randomized genomes using two different approaches and assessed whether similar trade-offs in tRNA abundance and mRNA secondary structure were still present (for more details on the randomization see ‘Materials and Methods’ section). By maintaining the codon usage in each individual gene, but randomizing the order within each sequence, we will assess whether the observed trade-offs naturally arise from the codon composition of the genome. The first randomization method shuffled the synonymous codon usage within each gene thereby keeping the amino acid sequence intact. The second randomization method shuffled all the codons in each gene, resulting in a changed amino acid and codon order, while the codon usage is maintained. Because the secondary structure of these randomized genomes will differ from the original, we computed their secondary structure using Vienna software package (33), see ‘Materials and Methods’ section. Comparisons were only made to the *E. coli* dataset in order to use a uniform approach when assessing the secondary structure landscape of mRNAs between the original gene set and the randomized counterpart.

**Figure 5.**
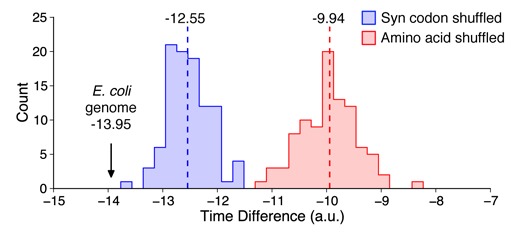
A strong selection pressure operates on the *Escherichia coli* genome to select for trade-offs between tRNA abundance and mRNA secondary structure. Time difference is calculated as the difference of the median predicted codon translation time between high and low structured regions. Greater negative values are evidence that these factors trade-off their contributions to smoothen translational speed. The time difference for *E. coli* is marked on the axis. The clear separation between both distributions and the native *E. coli* genome is significant in all cases.

We found that both randomization methods generated genomes with significantly reduced trade-offs between tRNA abundance and mRNA secondary structure (Figure 5; *P* = 3.9 × 10^−18^ in both cases, one-sample Wilcoxon test). Thus, the trade-offs cannot naturally arise from the codon bias present in the host suggesting that selection has operated to maintain this feature. A further interesting outcome of this analysis were the significant differences between the randomization methods themselves. Specifically, upon synonymous codon shuffling the trade-off was reduced in comparison to the amino acid shuffling method (*P* = 3.2 × 10^−32^, Mann–Whitney test). This suggests that synonymous codon choice and the amino acid sequence play a significant role in ensuring such a trade-off is present and offers two mechanisms by which such a trade-off can be developed and maintained.

### Trade-offs between tRNA abundance and mRNA structure improves gene expression

The selective pressures we observed suggest that the trade-offs made between tRNA abundance and mRNA secondary structure may play an important functional role. To explore this in the context of protein expression, we analyzed an existing experimental data in which 30 synonymous codon variants of a Φ29 DNA polymerase gene were synthesized and expressed under a strong promoter in *E. coli* (36). Each of these gene variants produced an identical protein product. However, the varying synonymous codon usage led to changes in the translation elongation dynamics due to differing tRNA abundance and propensity of each codon to participate in secondary structure along the transcripts. Applying the same approach, we compared the predicted local mRNA secondary structure with the tRNA abundance for codons in high and low structured regions for each gene independently. We reasoned that, if such trade-offs help smoothen translational speed and enable more efficient protein expression, we expect that greater deviations in tRNA abundance between high and low structured regions will lead to higher protein yields.

**Figure 6.**
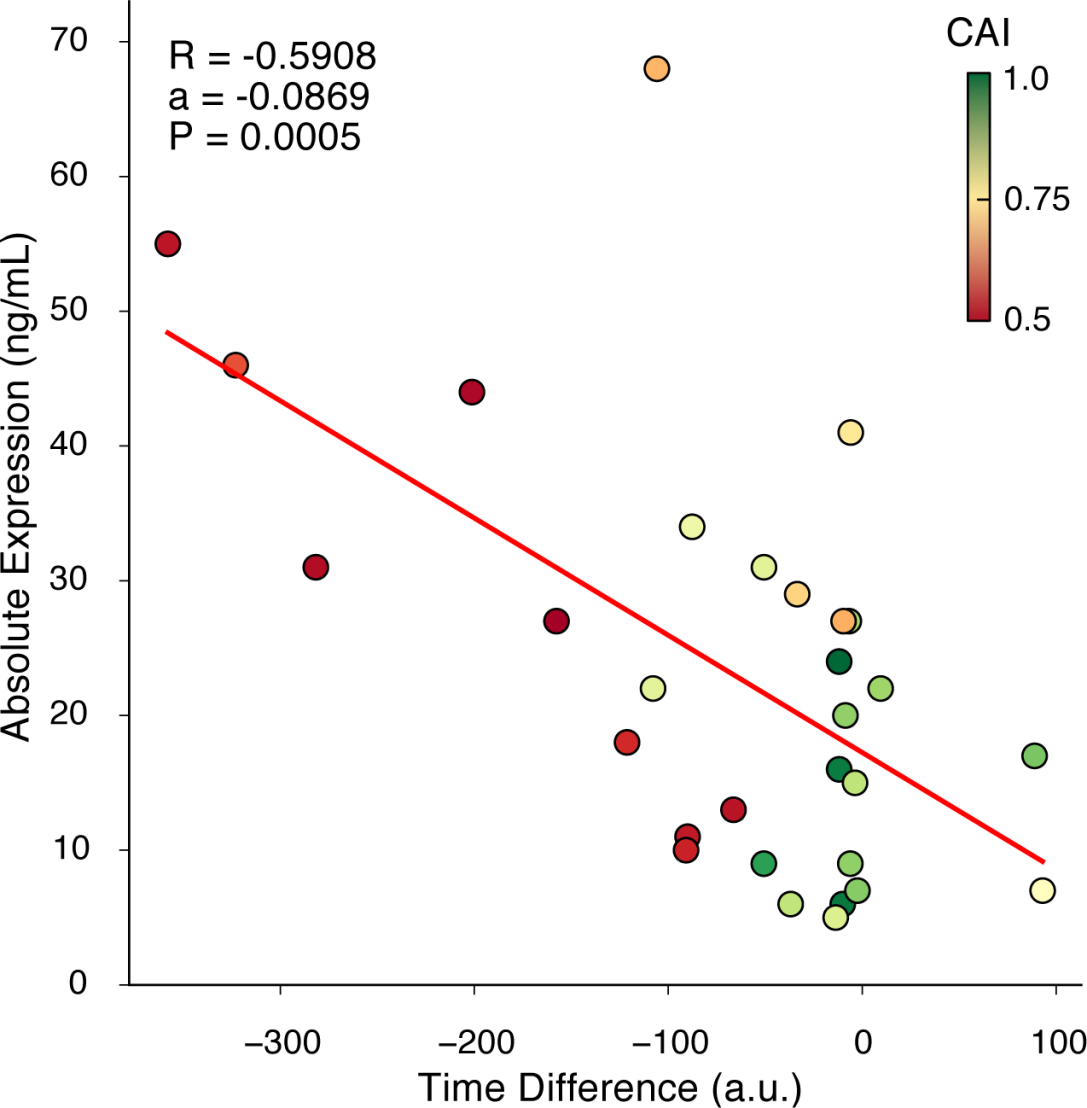
Synonymous codon choice in a synthetic gene effects expression level. Time difference is calculated as in Figure 5 as the difference in predicted codon translation time between high and low structured regions. Greater negative values correspond to stronger trade-offs between tRNA abundance and mRNA secondary structure which exhibits a significant negative correlation (*R* = −0.5908, *P* = 0.0005) with the absolute protein expression. The red line represents a linear least squares regression fit (*a* = −0.0869); points are colored (red = low, green = high) and labeled in relation to the codon adaptation index (CAI) of each variant.

Thus, we next calculated the time difference between the median codon translation time in high and low structured regions. Greater negative values mean that highly structured regions exhibited an increased bias toward faster translated codons (greater tRNA abundance), while low structured regions showed increased bias toward slower translated codons (lower tRNA abundance), implying that these factors trade-off their contributions to smooth overall translational speed. We found a significant negative correlation between the predicted time difference in codon translation times and the experimentally measured absolute protein expression (Figure 6; *R* = −0.5908, *P* = 0.0005), suggesting that smoothing of translation rate along a transcript plays an important role in improving protein expression.

For all gene variants the same promoter and ribosome binding site (RBS) was used for expression to minimize potential contextual effects that can influence transcription or translation. However, interactions between the RBS and the start of the coding sequence can significantly alter translation initiation rates (2,3). To assess whether the increased expression we observed was due to such interactions we used the RBS Calculator (2) to predict translation initiation rates. This analysis revealed no relationship between the predicted initiation rate and the protein expression level, suggesting that the effects we observe are solely due to translation elongation in the coding region. This finding is supported by recent work showing that translation initiation contributes minimally (∼1%) to expression variability of endogenous genes, compared to a much larger contribution (12%) for factors related to translation elongation (41). Furthermore, only in cases where secondary structure between the RBS and the start of a gene are high (rare for endogenous genes) (3), such interactions significantly influence expression (36).

We also investigated whether other measures of translational efficiency might explain the relationship we observed. The codon adaptation index (CAI) is a common measure often used to predict the efficiency of translation of each gene in the context of a particular host (42,43). It considers the codon usage in a set of highly expressed endogenous genes and compares this to the codon usage in the gene of interest. Values range from 0 to 1, with 1 representing a gene with perfect adaptation to the hosts’ codon usage pattern with predicted high expression, while 0 represents a poorly adapted gene with predicted low expression. By overlaying the CAI values on top of our existing results (see point color in Figure 6), it can be seen that while there is no clear trend between CAI and protein expression (36). However, we found that those variants with greatest expression tend to have the lower CAI values. Because CAI values are based purely on codon usages and considers an average across the entire gene, the measure is unable to account for potential local contextual effects that might affect a codons suitability and translation speed (44).

## DISCUSSION

Genome-scale analysis shows that a clear relationship between tRNA abundance and local mRNA secondary structure is maintained through codon choice across both *E. coli* and *S. cerevisiae* genomes. We find that regions of high secondary structure contain codons read by high-abundance tRNAs, while regions of low secondary structure contain codons pairing to tRNAs with lower abundance. Given that each of these factors in isolation significantly influences the speed of translation elongation (23,24), the relationship we observe suggests that a trade-off between their beneficial and detrimental effects smooths overall translation rate.

The rate of elongation of a single codon is a sum of several discrete processes, each of which depends on the specific codon identity and each differently contributing to the overall speed of decoding. These include: (i) processes outside the ribosome, e.g. aminoacylation of tRNAs by the cognate aminoacyl-tRNA synthetases, complex formation with elongation factor and delivery by diffusion to the ribosome (45) and (ii) decoding processes at the ribosome, e.g. sampling and accommodation of the correct tRNA, peptide bond formation and translocation from the A- to P-site (15). Detailed kinetic data from elaborated *in vitro* experiments show that among the 20 proteinogenic amino acids, the amino acid proline is incorporated into the nascent chain 3–6 times slower than the other 19 amino acids (17,46). The cyclic sterics of proline affects translation rates at the peptidyl transfer step (17) and proline has been associated with slow translation in ribosome profiling experiments (28). New developments in ribosome profiling that produce unbiased data on the position of ribosome fragments without using antibiotics to stall elongating ribosomes, suggest rather marginal differences in the residence time of the ribosome at different codons *in vivo* (16,18). In general, the residence time of the ribosome at different codons when they are in the A-site differs by a factor of ∼2, with GC-rich codons decoded at the slowest rate (16). Furthermore, some slow translated regions, e.g. codons at the 5′ end of the coding sequence or in the proximity of Shine-Dalgarno-like sequences in prokaryotes, suggest that other factors unrelated to the codon identity can slow translation (18,22). Overall, the influence of the cellular tRNA concentration on elongation of the single codons is the highest (16,20). Notably, proline codons, which are also GC-rich are among the slowly translated ones, but only consecutive proline codons cause ribosomal delays similar to the rare codon-specific delays (16). The latter are much rarer in the genomes and may only influence the elongation speed of specific transcripts. Thus, at global scale the active tRNA concentration (i.e. aminoacyl-tRNA) is a good approximation of the ribosome residence time and consequently the rate of elongation of each codon *in vivo*; other discrete steps at the ribosome have rather a marginal effect on the overall speed.

The reduction of excessively fast or slow translation rates along an mRNA would be beneficial to increase the rate of protein synthesis. Regions of excessively slow translational speed can lead to the formation of bottlenecks and cause ribosome collisions and potential queuing or pausing that may in turn trigger stress responses that prematurely recycle stalled ribosomes (47–49). Such problems have been highlighted in models designed to optimize expression rates, with non-uniform translational speed leading to an increased chance of ribosome collisions that reduce overall expression (50). However, these models have not considered the impact of the mRNA secondary structure in modulating the local translational speed. By assessing the observed trade-off in a library of synonymous codon variants, we find a significant trend whereby expression is maximized when a strong trade-off is present.

A less intuitive aspect of the benefit provided by a smoothed translation rate is why an increased translational speed in regions of low secondary structure is not beneficial to protein yield. From a purely translation-based perspective, an increased rate of elongation should directly improve efficiency. It is therefore puzzling why selection for less abundant tRNAs in this case would help. This confusion can be addressed by considering mRNA degradation. Recent experimental studies have shown that ribosomes stabilize mRNAs by shielding it from degradation (51,52) and models that incorporate dynamic shielding of mRNAs during co-transcriptional translation more accurately capture the observed *in vivo* decay characteristics (53). Thus, while transcripts with codons with high tRNA abundance and low mRNA structure would be most efficiently translated; the higher translation rate correlates with a lower ribosome density, which in turn increases the mRNA susceptibility to degradation. Optimal protein production will therefore arise from a complex trade-off between the many different processes at work during protein biogenesis and maximization of a single attribute may not always lead to a predictable or desired outcome.

The non-random nature of mRNA sequences has long been appreciated, with studies of randomized and shuffled genomes and mRNAs showing selection for a variety of features, e.g. the amino acid composition of proteins (54), mRNA secondary structure (55) (although this has been contested (56)) and synonymous codon choice across organisms (42,43) and within transcripts coding for genes with differing functions (57–59). Non-random preferences are also present in the use of codon pairs across genomes (60), potentially due to the large differences that are observed experimentally in their decoding speeds due to the specific codons found up and downstream—referred to ‘codon context’ (44). Moreover, many of these biases are not uniform across mRNAs, but localized to specific regions that influence the correct folding of the cognate protein, facilitate translation initiation due to reduced secondary structure at the start of a transcript (30,59), have correlated ordering of synonymous codons to ensure efficient recycling of tRNAs (61) and pause ribosomes through the formation of strong secondary structures to aid in the accurate decoding of codons at functionally critical points (62).

Our results highlight that the separated analysis of the impact of factors that alter translational speed, can miss potentially important relationships that are maintained between several factors at once. The ability for tRNA abundance and local mRNA secondary structure to trade-off their detrimental and beneficial influences on elongation rate means that their individual role cannot be disentangled. Only when viewed in the context of the entire process and with an understanding of their potential individual effects can we deliver valid interpretations of the data. For this reason, our results may help to resolve a puzzling finding from a genome-wide study of translation rate in *E. coli*, where studying the effect of many different modulators of translational speed in isolation showed only amino acid charge of the translated codon as having a measurable effect on translational speed (26). Furthermore, the connection we find suggests that many of the isolated relationships between factors such as secondary structure or synonymous codon choice and the non-random biases embedded within mRNA sequences, may be strengthened when considered in unison. For example, the role of these combined aspects on pausing sites for co-translational folding has yet to be analyzed, with our results suggesting that a pause may be better characterized by a combined negative effect on translational speed, not only their individual contribution.

Related to this, our work may also offer some insight into results from recent efforts in synthetic biology to perform large-scale synonymous re-coding of genomes. Lajoie *et al*. showed that it was possible to extensively recode using synonymous codons, 42 highly expressed genes in *E. coli* (63). Although reduced fitness (growth) was generally observed, the cells often remained viable. However, there were several cases in which specific synonymous codon substitutions had a significant impact, resulting in unpredictable responses. The relationship we have presented here may help to explain these complex failure modes as mRNA secondary structure and tRNA abundance can act either synergistically or antagonistically. For example, high secondary structure coupled with low tRNA abundance could lead to amplified pausing of ribosomes. For highly expressed genes this could lead to a severe sequestering of ribosomes that in extreme conditions could hinder the expression of other endogenous genes required for maintenance and growth. Validation of this hypothesis will be the focus of future work.

Optimization of gene sequences is important for improving the yield of protein products. To date most methods have relied on optimization of global transcript features such as reduced secondary structure across the entire transcript or optimal codon selection (13,14,36,43). However, such approaches often fail highlighting the need to address the coordinated impact of different factors on translation speed to improve expression. More recently, attempts have been made to model the translational process in greater detail, accounting for the influence of local effects along a transcript (50). However, these are still often limited to focusing on a single determinant of translational speed such as tRNA abundance or ordering constraints on the synonymous codons used (61). Our work suggests a potential extension to these models such that optimization is performed based on the influences of two factors with the aim of smoothing the overall translation rate, not maximizing it. Moreover, it provides a rational and universal tool for tuning expression of genes where certain secondary structures in the mRNA are unavoidable due to constraints on the amino acid sequence.

Although under normal conditions concentrations of charged tRNAs remain fairly constant, it has been shown experimentally that tRNA abundance can vary for differing growth rates (64) and amino acid starvation conditions (65). It is thought that such changes in availability may play a role in maintaining efficient protein synthesis by matching the varying demands placed on particular tRNA pools due to fluctuations in transcript levels (66) or by enabling a tuning of gene expression based on their functional role during a stress response (7,57,58). Furthermore, there is growing evidence that the dynamic modification of tRNAs can also impact translation elongation speed of cognate codons to efficiently tune their dynamics or potentially be the cause of disease (67). Although such regulation of translation was outside the scope of this study, it provides an interesting future direction.

In summary, this study provides the first evidence of a strong synergistic link between separate factors influencing translation in prokaryotic and eukaryotic organisms. This suggests that tRNA abundance and mRNA secondary structure together impose an important constraint on codon choice in many organisms. As our knowledge of how such mechanisms influence translation develops, piecing together how these are integrated to control various aspects of this process will form an integral part in fully understanding how cells use such modulators to refine their proteomes and will offer new methods for optimizing protein expression.

## SUPPLEMENTARY DATA

Supplementary Data are available at NAR Online.

SUPPLEMENTARY DATA
