# Supplementary material for: Trade-offs between tRNA abundance and mRNA secondary structure support smoothing of translation elongation rate
Source: Nucleic Acids Res. 2015 Mar 12;43(6):3022–32. doi: 10.1093/nar/gkv199 (PMC4381083; doi:10.1093/nar/gkv199)
Supplement: SUPPLEMENTARY DATA [file supp_43_6_3022__index.html]

Trade-offs between tRNA abundance and mRNA secondary structure support smoothing of translation elongation rate — Trade-offs between tRNA abundance and mRNA secondary structure support smoothing of translation elongation rate — SUPPLEMENTARY DATA 

# Trade-offs between tRNA abundance and mRNA secondary structure support smoothing of translation elongation rate

## SUPPLEMENTARY DATA

**Files in this Data Supplement:**

- SUPPLEMENTARY DATA
