# Supplementary material for: Trade-offs between tRNA abundance and mRNA secondary structure support smoothing of translation elongation rate
Source: Nucleic Acids Res. 2015 Mar 12;43(6):3022–32. doi: 10.1093/nar/gkv199 (PMC4381083; doi:10.1093/nar/gkv199)
Supplement: SUPPLEMENTARY DATA [file supp_gkv199_nar-03574-r-2014-File002.pdf]

Supplementary Information:
Trade-offs between tRNA abundance and mRNA secondary structure support smoothing of translation elongation rate

Thomas E. Gorochowski[1,2], Zoya Ignatova[3,4], Roel A.L. Bovenberg[1], and Johannes A. Roubos[1]

[1]DSM Biotechnology Center, P.O. Box 1, 2600 MA Delft, The Netherlands
[2]Current address: Department of Biological Engineering, Massachusetts Institute of Technology, Cambridge, USA
[3]Department of Biochemistry, Institute of Biochemistry and Biology, University of Potsdam, Potsdam-Golm, Germany
[4]Biochemistry and Molecular Biology, Department of Chemistry, University of Hamburg, Hamburg, Germany

Contents

Supplementary Figures **2**
Figure S1 Predicted codon translation time distributions for highly expressed mRNAs in *E. coli* 2
Figure S2 Codon usage of highly expressed genes for high and low structured regions in *E. coli* 3
Figure S3 Predicted codon translation time distributions for differing length mRNAs 4
Figure S4 Amino acid bias in regions of mRNA with high and low secondary structure 5

Supplementary Tables **6**
Table S1 Predicted translation time of codons based on tRNA availability 6
Table S2 GO enrichment for low structured regions with long predicted translation times 7

References **8**

Supplementary Figures

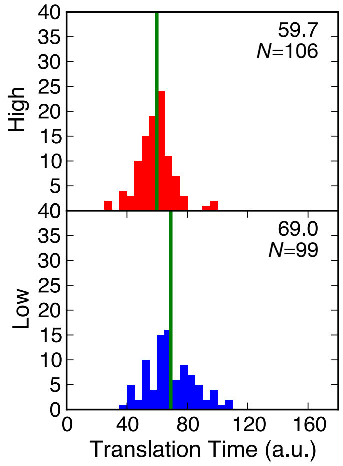

Figure S1: **Predicted codon translation time distributions for highly expressed mRNAs in *E. coli***. Upper plots (red) show regions with high mRNA secondary structure and lower plots (blue) show regions with low mRNA secondary structure. All regions analyzed had a minimal region length of 20 bp (for details see Methods section). Green vertical lines and value in top right corner of each plot denote the median value for the distribution. Set of highly expressed genes (N=255) was taken from HEG-DB [1]. Each bin has a width of 5 a.u.

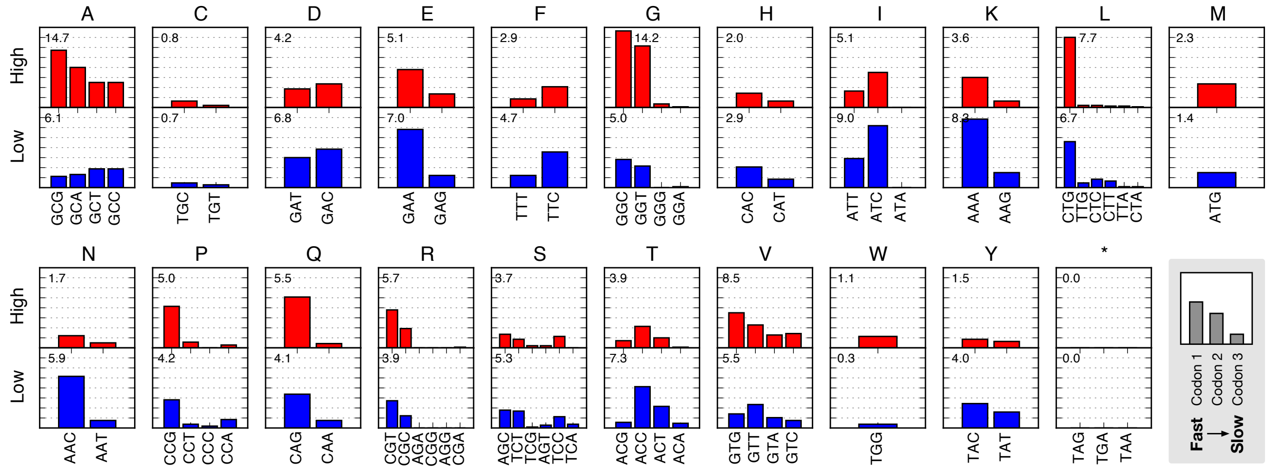

Figure S2: **Codon usage for protein coding regions of highly expressed mRNAs with high and low mRNA secondary structure across the *E. coli* genome**. Separate plots are displayed for each amino acid, with upper plots (red) showing regions with high mRNA secondary structure and lower plots (blue) regions with low mRNA secondary structure. Each plot displays the normalized usage of each codon for the associated amino acid on a scale from 0 to 0.08 and codons are sorted from left to right in terms of predicted translational times, see Table S1 (stop codons are excluded), fastest (left) to slowest (right).

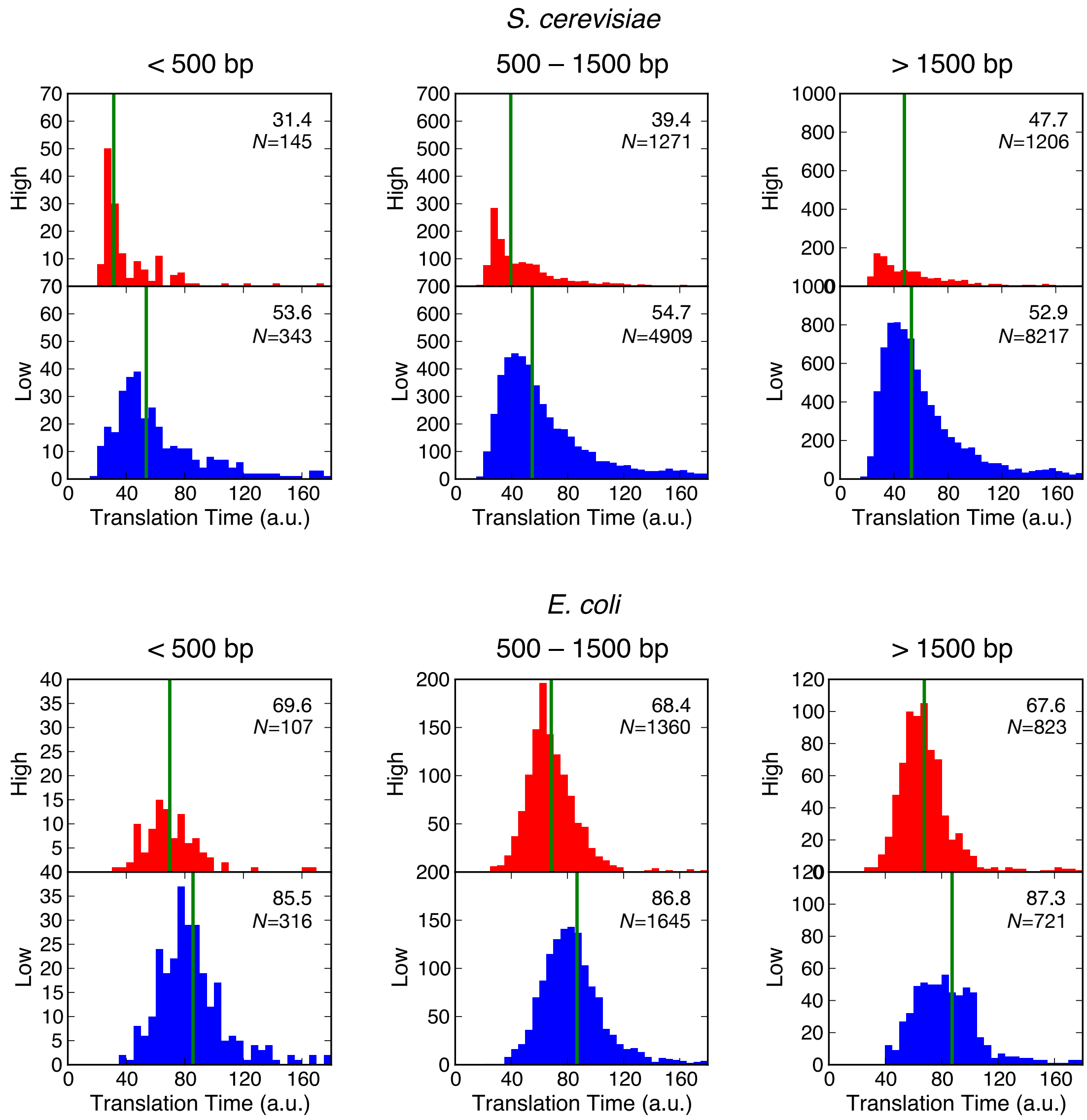

Figure S3: **Predicted codon translation time distributions for differing length mRNAs in the *S. cerevisiae* and *E. coli* genomes**. Upper plots (red) show regions with high mRNA secondary structure and lower plots (blue) show regions with low mRNA secondary structure. All regions analyzed had a minimal region length of 20 bp (for details see Methods section). Green vertical lines and value in top right corner of each plot denote the median value for the distribution. Each bin has a width of 5 a.u.

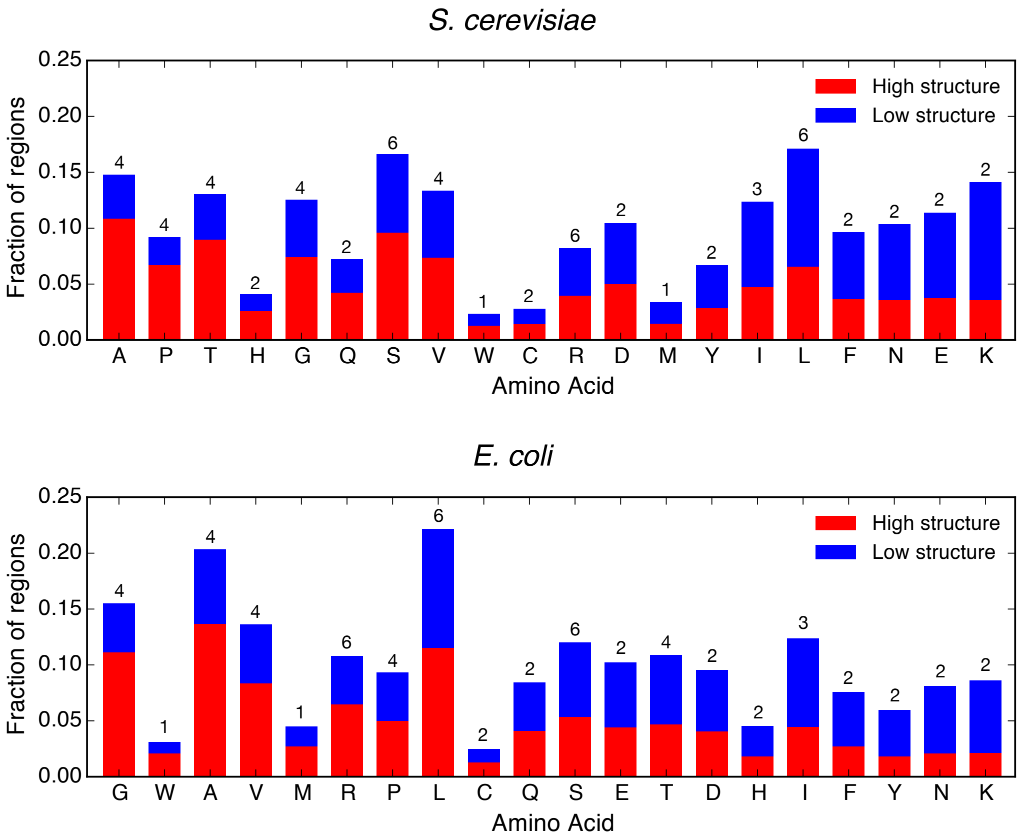

Figure S4: **Amino acid bias in regions of mRNA with high and low secondary structure**. Amino acids have been sorted by their bias towards highly structured regions: (left) larger ratio of high to low structured regions, to (right) larger ratio of low to high structured regions. Fractions of low and high structured regions each sum to 1 independently. Number of synonymous codons coding for each amino acid are included above each bar. Note the higher diversity in potential synonymous codon choice for amino acids with a greater propensity to be found in highly structured regions.

Supplementary Tables

Table S1: **Predicted translation time of codons based on tRNA availability**. Times are in arbitrary units and taken from previous studies by Reuveni *et al.* for *S. cerevisiae* [2] and Zhang *et al.* for *E. coli* [3].

		Translation Time				Translation Time	
**Amino Acid**	**Codon**	*S. cerevisiae*	*E. coli*	**Amino Acid**	**Codon**	*S. cerevisiae*	*E. coli*
A	GCT	24.8	89.3	P	CCT	136.5	101.0
A	GCC	34.5	105.3	P	CCC	189.6	188.7
A	GCA	54.6	67.6	P	CCA	27.3	5000.0
A	GCG	170.6	40.8	P	CCG	85.3	56.2
C	TGT	155.5	129.9	Q	CAA	30.3	84.7
C	TGC	68.3	59.2	Q	CAG	70.4	73.5
D	GAT	38.9	42.9	R	CGT	45.5	24.8
D	GAC	17.1	71.9	R	CGC	63.2	36.6
E	GAA	19.5	16.3	R	CGA	1365.0	163.9
E	GAG	42.1	84.0	R	CGG	273.0	101.0
F	TTT	62.2	123.5	R	AGA	24.8	74.6
F	TTC	27.3	126.6	R	AGG	60.4	153.8
G	GGT	38.9	32.5	S	TCT	24.8	73.5
G	GGC	17.1	27.2	S	TCC	34.5	149.3
G	GGA	91.0	71.9	S	TCA	91.0	222.2
G	GGG	92.2	52.1	S	TCG	139.3	80.6
H	CAT	88.8	256.4	S	AGT	310.9	129.9
H	CAC	39.0	166.7	S	AGC	136.5	70.9
I	ATT	21.0	29.9	T	ACT	24.8	106.4
I	ATC	29.2	64.9	T	ACC	34.5	74.6
I	ATA	136.4	204.1	T	ACA	68.2	303.0
K	AAA	39.0	44.2	T	ACG	119.7	66.2
K	AAG	16.8	140.8	V	GTT	19.5	50.8
L	TTA	39.0	185.2	V	GTC	27.1	112.4
L	TTG	22.3	24.8	V	GTA	136.4	84.7
L	CTT	621.9	158.7	V	GTG	103.4	35.6
L	CTC	273.0	120.5	W	TGG	45.5	68.5
L	CTA	91.0	2000.0	Y	TAT	77.7	79.4
L	CTG	284.4	12.6	Y	TAC	34.1	53.2
M	ATG	27.3	24.5	*	TGA	—	294.1
N	AAT	62.2	119.0				
N	AAC	27.3	99.0				

Table S2: **GO enrichment for genes containing low structured mRNA regions with long predicted translation times in *S. cerevisiae*.** Slowly translated regions are defined as having an average translation time >140 a.u. and GO enrichment calculated using AmiGO [4] with $P < 0.01$.

GO Slim Term		N
**Biological Process**		
GO:0044699	single-organism process	457
GO:0044763	single-organism cellular process	411
GO:0051179	localization	209
GO:0051234	establishment of localization	193
GO:0006810	transport	188
GO:0044765	single-organism transport	160
GO:0071702	organic substance transport	136
GO:0051641	cellular localization	135
GO:0033036	macromolecule localization	130
GO:0051649	establishment of localization in cell	116
GO:0008104	protein localization	112
GO:0045184	establishment of protein localization	99
GO:0015031	protein transport	93
GO:0034613	cellular protein localization	82
GO:0071705	nitrogen compound transport	52
GO:0006997	nucleus organization	22
**Cellular Component**		
GO:0005623	cell	615
GO:0044464	cell part	615
GO:0043226	organelle	527
GO:0043229	intracellular organelle	526
GO:0043227	membrane-bounded organelle	496
GO:0043231	intracellular membrane-bounded organelle	495
GO:0044422	organelle part	373
GO:0044446	intracellular organelle part	372
GO:0016020	membrane	311
GO:0044425	membrane part	273
GO:0031224	intrinsic component of membrane	237
GO:0016021	integral component of membrane	234
GO:0031090	organelle membrane	204
GO:0012505	endomembrane system	148
GO:0098588	bounding membrane of organelle	145
GO:0031967	organelle envelope	95
GO:0031975	envelope	95
GO:0005773	vacuole	58
GO:0000322	storage vacuole	52
GO:0000323	lytic vacuole	52
GO:0000324	fungal-type vacuole	52
GO:0044437	vacuolar part	52
GO:0005774	vacuolar membrane	51
GO:0005635	nuclear envelope	35
GO:0031965	nuclear membrane	24
GO:0005643	nuclear pore	19
**Molecular Function**		
GO:0005215	transporter activity	80
GO:0017056	structural constituent of nuclear pore	14

References

[1] Puigbò,P., Romeu,A. and Garcia-Vallvé,S. (2008) HEG-DB: a database of predicted highly expressed genes in prokaryotic complete genomes under translational selection. *Nucleic Acids Res.*, **36**, D524-D527.

[2] Reuveni,S., Meilijson,I., Kupiec,M., Ruppin,E. and Tuller,T. (2011) Genome-Scale Analysis of Translation Elongation with a Ribosome Flow Model. *PLoS Comp. Biol.*, **7**, e1002127.

[3] Zhang,G. and Ignatova,Z. (2009) Generic algorithm to predict the speed of translational elongation: implications for protein biogenesis. *PLoS One*, **4**, e5036.

[4] Carbon,S., Ireland,A., Mungall,C.J., Shu,S.Q., Marshall,B., Lewis,S. and the AmiGO Hub and the Web Presence Working Group (2009) AmiGO: online access to ontology and annotation data. *Bioinformatics*, **25**, 288-289.
